# Colloidal Synthesis of MoSe_2_/WSe_2_ Heterostructure Nanoflowers via Two-Step Growth

**DOI:** 10.3390/ma14237294

**Published:** 2021-11-29

**Authors:** Yunjeong Hwang, Naechul Shin

**Affiliations:** 1 Program in Biomedical Science and Engineering, Inha University, Incheon 22212, Korea; ruflus16@kims.re.kr; 2 Program in Smart Digital Engineering, Inha University, Incheon 22212, Korea; 3 Materials Center for Energy Convergence, Korea Institute of Materials Science (KIMS), 797 Changwondaero, Sungsan-gu, Changwon 51508, Korea; 4 Department of Chemical Engineering, Inha University, Incheon 22212, Korea

**Keywords:** transition metal dichalcogenides, MoSe_2_, WSe_2_, heterostructure, core-shell, colloidal synthesis

## Abstract

The ability to control the active edge sites of transition metal dichalcogenides (TMDs) is crucial for modulating their chemical activity for various electrochemical applications, including hydrogen evolution reactions. In this study, we demonstrate a colloidal synthetic method to prepare core-shell-like heterostructures composed of MoSe_2_ and WSe_2_ via a two-step sequential growth. By overgrowing WSe_2_ on the surface of preexisting MoSe_2_ nanosheet edges, MoSe_2_-core/WSe_2_-shell heterostructures were successfully obtained. Systematic comparisons of the secondary growth time and sequential order of growth suggest that the low synthetic temperature conditions allow the stable overgrowth of shells rich in WSe_2_ on top of the core of MoSe_2_ with low Gibbs formation energy. The electrochemical analysis confirms that the catalytic activity correlates to the core-shell composition variation. Our results propose a new strategy to control the edge site activity of TMD materials prepared by colloidal synthesis, which is applicable to diverse electrochemical applications.

## 1. Introduction

Transition metal dichalcogenides (TMDs) have attracted tremendous attention owing to their interesting properties originating from their two-dimensional (2D) structures, which generally consist of a central transition metal layer sandwiched between chalcogen atomic layers. Along with the geometry, a diverse selection of constituents (metals and chalcogens), crystal phases (e.g., trigonal prismatic and octahedral), and a number of layers impart characteristic physical properties, depending on the TMD structures. As an example, group VI TMDs, including MoS_2_ or WSe_2_, primarily show semiconducting properties with a bandgap of −1 eV, while group V TMDs, such as the semimetallic VSe_2_ or NbS_2_ exhibit superconductivity [1,2]. The phase transition between metallic 1T and semiconducting 2H-phases occurs via chemical intercalation [3,4] or laser excitation [5], without changing the TMD layer constituents. Moreover, the optical bandgap of semiconducting TMD layers exhibits a direct-to-indirect transition depending on the number of layers.

More importantly, the dangling bond-free van der Waals (vdW) interactions, together with the analogous crystal structures, allow the preparation of TMD heterostructures with diverse compositions and geometries. The formation of alloyed TMDs with entire solid solution ranges has been demonstrated via tunable bandgap engineering [6,7]. Furthermore, vertically stacked and laterally extended heterostructures with variable optical/electronic properties have been reported [8,9]. Accordingly, numerous preparation techniques, including mechanical exfoliation or physical/chemical vapor deposition, have been employed to prepare TMD-based heterostructures [10,11,12,13].

While these methods lead to complex heterostructures with a high level of control from atomically sharp junctions to alloys with arbitrary compositions, their applications are limited by complicated preparation procedures and low scalability. Alternatively, solution-based synthetic approaches overcome these limitations with scalable growth protocols at relatively mild temperatures [14,15]. Furthermore, a homogenous reaction environment enables the formation of TMD nanosheets with radially outward oriented edges, which is advantageous for catalysis. Regarding the heterostructure formation, TMD catalytic performance is enhanced by preparing type II heterojunctions, because the electron-hole separation facilitates the charge transfer [16,17,18]. Nevertheless, growing core-shell like TMD heterostructures in the solution phase remains challenging because the thermodynamic control of stepwise nucleation is hindered in the homogeneous precursor mixture.

Here, we present a synthetic approach for the preparation of core-shell-like TMD nanoflower heterostructures based on colloidal crystal growth in the solution phase. We selected MoSe_2_ and WSe_2_ as model systems for the heterostructures because of their comparable lattice constants (2.7 Å for MoSe_2_ (100) and 2.8 Å for WSe_2_ (100)). The sequential colloidal growth using primarily grown MoSe_2_ nanosheets as the host material for the subsequent nucleation and growth of WSe_2_ resulted in the formation of phase-segregated, core-shell-like nanosheets. The systematic comparison of the heterostructure compositional distribution as a function of secondary growth time and growth order provides important information on the thermodynamic and kinetic factors dominating the growth. Specifically, low-temperature conditions and the Gibbs formation energy difference between MoSe_2_ and WSe_2_ enable the stable growth of MoSe_2_-core/WSe_2_-shell heterostructure nanosheets. Moreover, electrochemical measurements based on the hydrogen evolution reaction evaluate the catalytic activity correlation to the core-shell composition. These results suggest that tuning the active site density of colloidally synthesized TMD materials is a crucial strategy for various electrochemical applications.

## 2. Materials and Methods

### 2.1. Materials

Molybdenum hexacarbonyl (Mo(CO)_6_, 98%), tungsten hexacarbonyl (W(CO)_6_, 99.99 %), tetradecylphosphonic acid (TDPA, 97%), oleic acid (OA, technical, 90%), and diphenyl diselenide (Ph_2_Se_2_, 98%) were purchased from Sigma-Aldrich (Darmstadt, Germany). Solvents, including toluene (99.5%) and *n*-butanol (99%), were purchased from Duksan (Ansan, South Korea). All chemicals were used as received, without further purification.

### 2.2. Synthesis of MoSe_2_, WSe_2_, and MoSe_2_/WSe_2_ Heterostructures

MoSe_2_ and WSe_2_ nanosheets were grown using a colloidal hot-injection method. 108 mg (0.4 mmol) of Mo(CO)_6_ or 141 mg (0.4 mmol) of W(CO)_6_ were added into a 100 mL three-neck flask which was pre-loaded with 78.5 mg (0.28 mmol) of TDPA dissolved into 6 g of OA. 250 mg Ph_2_Se_2_, the selenium precursor, was dissolved in 4 g of OA in a separate vial. The Mo or W precursor mixtures were heated to 130 °C for 10 min and kept for 20 min under Ar atmosphere. The temperature was increased to 350 °C (for MoSe_2_) and 330 °C (for WSe_2_) at a rate of 4 °C/min, followed by the Se precursor solution injection. The nanosheet growth was maintained for 2 h, and then the flask was removed from the heating mantle to gradually cool down to room temperature. The crude solution was diluted with a solvent mixture composed of toluene and *n*-butanol in a 2:1 volume ratio. The suspension was then centrifuged for 30 min at 4000 rpm. The black powdery product was washed three times with a solvent mixture of toluene and *n*-butanol. Core–shell heterostructures were prepared by sequential growth of WSe_2_ on MoSe_2_ nanosheets or vice versa. The as-synthesized MoSe_2_ and WSe_2_ nanosheets were loaded into a 100 mL three-neck flask with 0.4 mmol of W(CO)_6_ and Mo(CO)_6_, respectively, and 78.5 mg of TDPA dissolved in 6 g of OA. The temperature profiles and the injection amount of the Se precursor were identical to those of the individual nanosheet growth, except that the secondary growth time varied from 30 min to 6 h.

### 2.3. Characterization

The as-grown nanosheets dispersed in toluene were drop-casted onto Si and SiO_2_/Si substrates, and their morphologies were examined using a scanning electron microscope (SEM, SU8010, Hitachi, Tokyo, Japan). The same substrates were used for the investigation of structures using a custom-built Raman spectrometer equipped with a charge-coupled device (DV420A-OE, Toshiba, Tokyo, Japan). The wavelength and power of the laser were 532 nm and 5 mW, respectively. The sample suspension was drop-cast onto a TEM grid (#01824, Ted Pella, Redding, CA, USA) for transmission electron microscopy (TEM, JEM2100F, JEOL, Tokyo, Japan). Microscopic images and selected area diffraction patterns were obtained under an operating voltage of 200 kV. The crystallinity and chemical composition of the samples were measured using powder X-ray diffraction (PXRD, X’Pert-PRO MRD, PANalytical, Malvern, United Kingdom) and X-ray photoelectron spectroscopy (XPS, K-Alpha, Thermo Scientific, Seoul Korea). Prior to the measurements, the samples were dried at 300 °C for 2 h under N_2_ atmosphere.

### 2.4. Electrochemical Measurements

The hydrogen evolution reaction (HER) was performed with a three-electrode-configuration HER potentiostat (PGSTAT 302N, Autolab, Utrecht, The Netherlands). Graphite rod and KCl-saturated Ag/AgCl were used as the counter and reference electrodes, respectively. The catalyst ink was prepared by mixing 4 mg of the catalyst (sample powders after drying), 30 μL of Nafion solution, and 1 mL of an ethanol-deionized water mixture (1:4 ratio). The mixed ink solution was ultrasonicated for 1 h. The catalyst ink (5 μL) was deposited onto a polished glassy carbon electrode and dried overnight. Linear sweep voltammetry measurements were conducted in 0.5 M H_2_SO_4_ solution with a sweep rate of 5 mV/s from 0.42 V to −0.78 V vs. reversible hydrogen electrode (RHE).

## 3. Results and Discussion

**Scheme 1:**
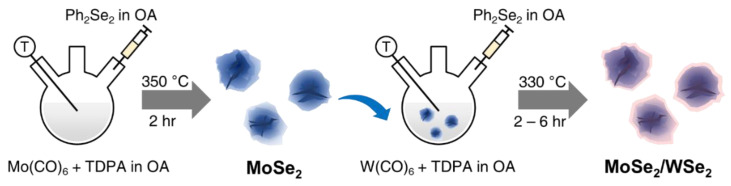
Schematic illustration of the synthetic process of MoSe_2_/WSe_2_ heterostructure via the two-step colloidal synthesis.

The MoSe_2_/WSe_2_ heterostructures were prepared via a sequential two-step colloidal synthesis, as illustrated in Scheme 1. First, MoSe_2_ nanosheets were primarily grown upon hot injection of diphenyl diselenide dissolved in oleylamine (OA) into a solution of Mo(CO)_6_ dissolved in OA with TDPA at 350 °C and reacted for 2 h. The as-grown MoSe_2_ nanosheets served as the substrate for the secondary growth of WSe_2_; therefore, MoSe_2_, W(CO)_6_, and TDPA were mixed in a three-neck flask, followed by a hot injection of diphenyl diselenide at 350 °C. After 2–6 h growth, the final MoSe_2_/WSe_2_ heterostructures were obtained.

**Figure 1:**
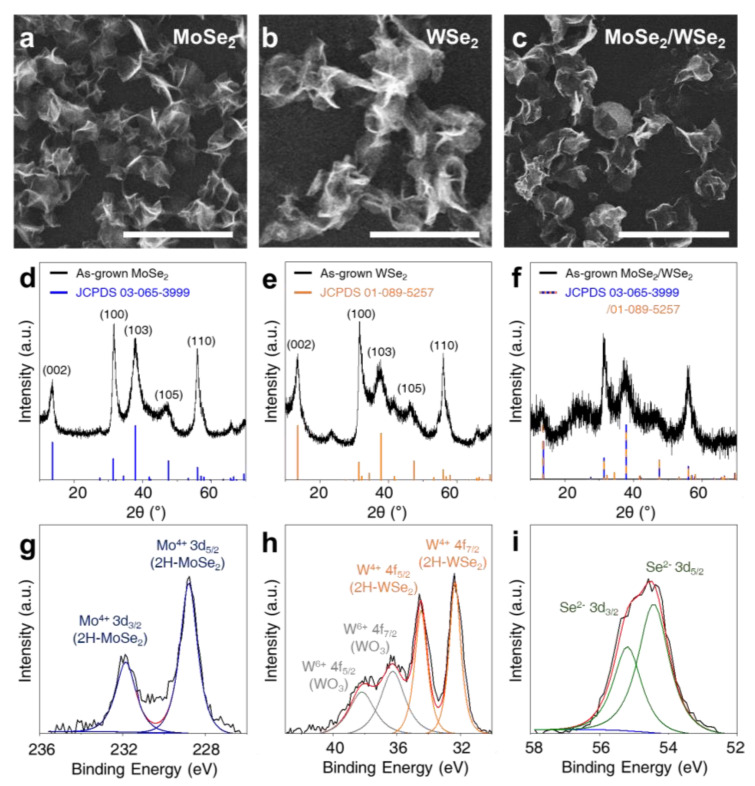
SEM images of the colloidally synthesized (**a**) MoSe_2_, (**b**) WSe_2_, and (**c**) MoSe_2_/WSe_2_ hetero-nanosheets. Scale bars, 1 μm. Powder XRD spectra obtained from (**d**) MoSe_2_, (**e**) WSe_2_, and (**f**) MoSe_2_/WSe_2_ nanosheets, shown together with the reference peaks from the corresponding JCPDS library (MoSe_2_: JCPDS 03-065-3999 and WSe_2_: JCPDS 01-089-5257). Core-level XPS spectra of (**g**) Mo 3d, (**h**) W 4f, and (**i**) Se 3d of MoSe_2_/WSe_2_ hetero-nanosheets.

Figure 1a–c illustrates the SEM images of the representative MoSe_2_, WSe_2_, and MoSe_2_/WSe_2_ heterostructures. The agglomerates of nanosheets with flower-like morphologies are confirmed from all the samples, analogously to previous studies [15,19]. The average diameter of the individual domains were in the range 200–300 nm. The powder XRD analysis shown in Figure 1d–f confirmed the correspondence of the agglomerated nanosheet diffraction pattern to the reference data (JCPDS #: 03-065-3999 (MoSe_2_), 01-089-5257(WSe_2_)). Five peaks located at 2θ = 13.6° (or 13.7°), 31.4°, 37.8° (or 37.9°), 47.4°, and 55.9° (or 56.0°) were assigned to the (002), (100), (103), (105), and (110) planes of MoSe_2_ (WSe_2_), respectively, indicating that the as-grown materials displayed the characteristic crystal structures of the 2D TMDs prepared via colloidal synthesis. Notably, since MoSe_2_ and WSe_2_ are characterized by comparable crystal structures (2H phase) and lattice constants [15,20], the as-obtained MoSe_2_/WSe_2_ hetero-nanosheets exhibited analogous morphologies and XRD peak patterns to the corresponding single TMD counterparts (Figure 1f). Despite the indistinguishable shapes and structures, XPS measurements of the MoSe_2_/WSe_2_ hetero-nanosheets (Figure 1g–i) confirmed the concurrent presence of both Mo and W. The peaks located at 229 and 232 eV (Figure 1g) corresponding to 2H MoSe_2_ (Mo^4+^ 3d_5/2_ and Mo^4+^ 3d_3/2_, respectively) suggested that Mo in the core region of the heterostructure largely retained its unoxidized state [21]. Conversely, the core-level W 4f spectrum of the same sample (Figure 1h) indicated the presence of both oxidized (WO_3_, W^6+^ 4f_5/2_ at 38 eV and W^6+^ 4f_7/2_ at 36 eV) and unoxidized (2H-WSe_2_, W^4+^ 4f_5/2_ at 35 eV and W^4+^ 4f_7/2_ at 33 eV) states [22]. The core-level Se^2−^ spectrum (Se^2−^ 3d_5/2_ at 54.5 eV and Se^2−^ 3d_3/2_ at 55.3 eV) shows no direct evidence of Se oxidation, as shown in Figure 1i [23]. The observed distinct oxidized states between Mo and W suggest a selective oxidation of the outer regions, rich in WSe_2_, whereas the inner domains, primarily consisting of MoSe_2_, were preserved. Therefore, the results presented in Figure 1 provide initial evidence of the uneven distribution of Mo and W in the MoSe_2_/WSe_2_ heterostructures.

**Figure 2:**
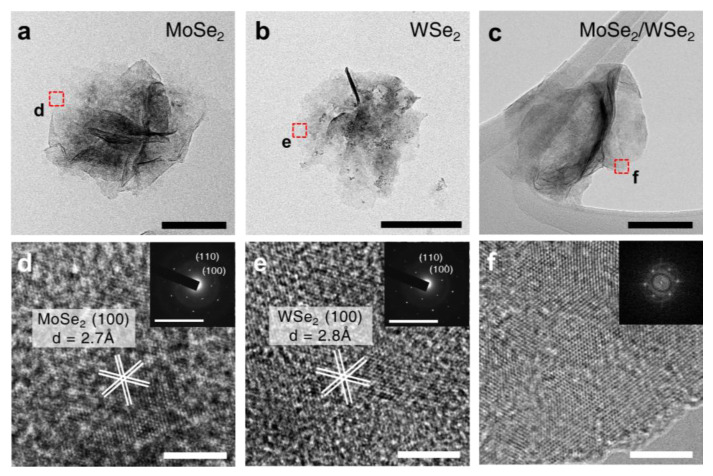
(**a**–**c**) Low-magnification bright field TEM images of (**a**) MoSe_2_, (**b**) WSe_2_, and (**c**) MoSe_2_/WSe_2_ hetero-nanosheets. Scale bars: (**a**,**b**) 200 nm and (**c**) 300 nm. (**d**–**f**) HRTEM images corresponding to the regions highlighted by dashed red boxes shown in (**a**–**c**) Scale bars: (**d**,**e**) 5 nm and (**f**) 20 nm. Insets in (**d**–**f**) correspond to the SAED and FFT patterns. Scale bars, 100 nm^−1^.

TEM measurements were conducted to investigate the as-grown-nanosheet crystal structure. Figure 2a–c display low-magnification TEM images of individual MoSe_2_, WSe_2_, and MoSe_2_/WSe_2_ nanosheets, respectively, confirming their nanoflower-like morphologies with noticeable thin edges. High-resolution TEM (HRTEM) images obtained near the edges of each sample (highlighted by dashed red boxes) are shown in Figure 2d–f. Lattice fringes spaced by 2.7 Å (Figure 2d) and 2.8 Å (Figure 2e), corresponding to the (100) planes of MoSe_2_ and WSe_2_, showed six-fold symmetry [15,20]. The selected area electron diffraction (SAED) patterns in the insets exhibited clear diffraction spot separation with the zone axis along the [001] direction, confirming that the edges of both MoSe_2_ and WSe_2_ are primarily composed of highly crystalline monolayers. While similar six-fold symmetric lattice fringes were observed for the MoSe_2_/WSe_2_ heterostructures, the fast Fourier transform (FFT) pattern (inset of Figure 2f) was stretched along the edges, reflecting the presence of curved monolayers facing outwards.

**Figure 3:**
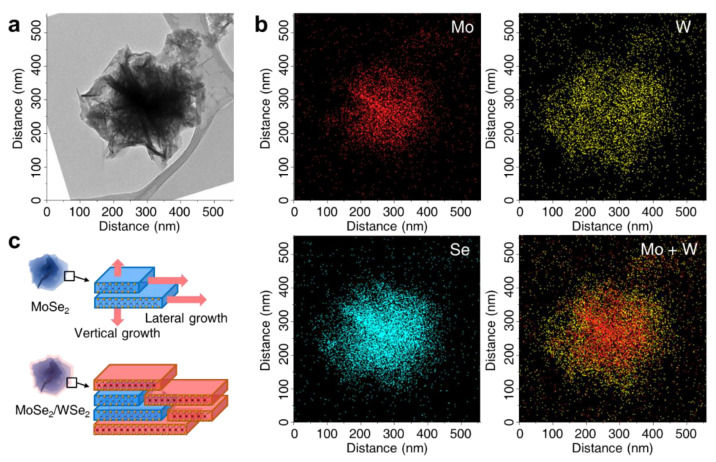
(**a**) Low-magnification TEM image of a MoSe_2_/WSe_2_ heterostructure and (**b**) corresponding EDS elemental maps of Mo, W, and Se in the heterostructure. The bottom right panel of (**b**) is a superimposed image of Mo and W spatial distribution. (**c**) Schematic illustration showing the heterostructure formation via vertical and lateral growth of WSe_2_ layers on the preexisting MoSe_2_ edge surface.

Figure 3a,b show the energy-dispersive X-ray spectroscopy (EDS) measurements of a representative MoSe_2_/WSe_2_ heterostructure. The elemental mappings of Mo, W, and Se (Figure 3b) confirmed that the spatial distributions of Mo and W are distinct in a 350 nm-sized hetero-nanosheet. While the signals of Mo are prominent only within the core region (200 nm), those of W appear uniformly distributed across all the domains (350 nm). The spatially distinct distributions of Mo and W were further validated by superimposing the mapping images (lower right panel of Figure 3b). Hence, the MoSe_2_/WSe_2_ hetero-nanosheets prepared via the two-step method exhibited core-shell-like structures. As the SEM and TEM images of MoSe_2_ and WSe_2_ in Figure 1 and Figure 2 indicate that the as-grown individual nanosheets exhibit three-dimensionally connected planar edges, we speculate that the sequential growth will result in the formation of WSe_2_ secondary layers both on top of the planes and along the edges of MoSe_2_, as illustrated in Figure 3c.

**Figure 4:**
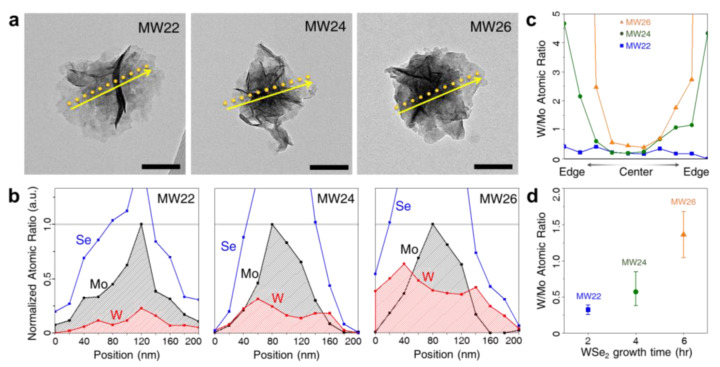
(**a**) TEM images of the MoSe_2_/WSe_2_ heterostructures prepared from different WSe_2_ secondary growth times (2, 4, and 6 h) on the primary MoSe_2_ nanosheets pre-grown for 2 h. Scale bars, 100 nm. (**b**) Corresponding elemental ratios of Mo, W, and Se obtained from the point EDS spectra measured across the nanostructure domains. The point arrays of the measurements are highlighted by the yellow dots in (**a**). All the ratios were normalized to the positions with the highest Mo concentration (highlighted by the horizontal gray lines). (**c**) Profiles of W/Mo atomic ratio according to the relative positions from the domain center. (**d**) W/Mo atomic ratios as a function of the secondary WSe_2_ growth time.

To corroborate the core-shell-like growth of WSe_2_ on MoSe_2_, we systemically investigated the distribution of W in the heterostructures as a function of the secondary growth time. Three samples were prepared by varying the secondary growth times, as shown in the TEM images in Figure 4a. Here, MW22, MW24, and MW26 denote the heterostructures of WSe_2_ grown for 2, 4, and 6 h, respectively, over the primary MoSe_2_ nanoflowers obtained after 2 h of growth. The profiles of W:Mo and Se:Mo atomic ratios corresponding to the 11 linear yellow dots crossing each nanoflower are shown in Figure 4b. For comparison, the ratios were normalized to the point with the highest Mo concentration. Notably, W atomic ratio increased in the outer domain region with the secondary growth time, consistently with the core-shell-like growth of WSe_2_. Figure 4c shows the W:Mo ratio distribution across the nanoflower domains. While the ratio near the outer edges drastically increased with the growth time, near the center it remained lower than 1. The change in the overall W:Mo ratio of each sample (Figure 4d) further corroborated the increase in W concentration proportionally to the secondary growth time. Remarkably, WSe_2_ layer heterogeneous nucleation was favored on the surface of the pre-grown MoSe_2_ domains for the core-shell structures observed in this study. The activation energy barrier for WSe_2_ nucleation on the preexisting MoSe_2_ surface was lower than that for homogeneous nucleation because of the reduced surface free energy [24]. Since the individual WSe_2_ nanosheets were absent in the samples grown via this two-step growth, it is speculated that the core-shell-like growth was favored over the independent nucleation, especially considering the maximum growth temperature of colloidal synthesis of 400 °C.

**Figure 5:**
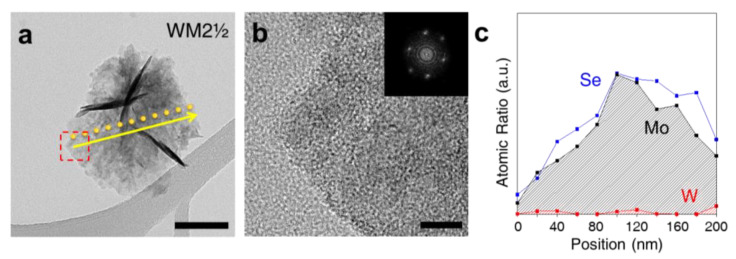
(**a**) Low-magnification TEM image of a nanostructure obtained from 30 min MoSe_2_ secondary growth on the WSe_2_ nanosheets pre-grown for 2 hrs. Scale bar, 100 nm. (**b**) HRTEM image measured at the edge region highlighted by the dashed red box in (**a**). The inset indicates the corresponding FFT pattern. Scale bar, 10 nm. (**c**) Elemental ratio profiles of Mo, W, and Se across the nanostructure, obtained from the point arrays highlighted by the yellow dots in (**a**).

While the formation of the phase-segregated structures was preferred to the mixed alloys under our selected growth conditions, the difference in the formation enthalpy between MoSe_2_ and WSe_2_ may affect the spatial distribution of the components (i.e., core vs. shell), according to the chronological order of the growth protocol. Figure 5 displays the results of the heterostructure growth in reverse order for WSe_2_ growth for 2 h, followed by MoSe_2_ growth for 30 min (marked as WM2½). While both TEM and HRTEM images indicate that the nanosheet size and the monolayer edge configurations are similar to those of the MW samples (Figure 5a,b), the elemental analysis across the domain points out striking differences. The concentration of W was substantially lower than that of Mo, and only a small amount was detected near the edge (Figure 5c), indicating that W in the primary nanosheet structure can dynamically interchange with Mo during the secondary growth.

Two possible explanations of the observed Mo-rich nanosheets from the reverse order growth can be considered. First, the MoSe_2_ nanosheet particles grew homogeneously without the pre-grown WSe_2_ surface assistance. According to this assumption, both MoSe_2_ and WSe_2_ independently exist in the final product. However, no WSe_2_ nanosheets were observed in this sample, suggesting that the hypothesis of homogeneous nucleation can be excluded. The second suggested scenario considers the substitution via interdiffusion of W in the primarily-grown-WSe_2_ nanosheet by Mo during the second step. The lower Gibbs free energy for the MoSe_2_ formation compared to WSe_2_ under the selected growth temperature (<700 °C) [25] suggests that MoSe_2_ tends to reduce its surface energy relatively to WSe_2_. Thus, Mo diffused towards the nanosheet core upon shell growth on the preexisting WSe_2_ nanosheet surfaces. Noticeably, the formation of the Mo_1−x_W_x_Se_2_ alloy was unlikely because of the low-temperature conditions used in colloidal synthesis, with a consequent reduction of the entropic contribution to the formation energy of mixed alloys. A similar behavior was observed in the two-step sequential growth of the WS_2_/MoS_2_ heterostructure employing the chemical vapor deposition (CVD) technique, as reported by Bogaert et al. [26]. In that case, the sequential growth of WS_2_-MoS_2_ showed the formation of Mo_0.5_W_0.5_S_2_ at high temperatures (>680 °C), whereas the MoS_2_ core-WS_2_ shell heterostructure was found at a low temperature (650 °C), which was reversed from the growth order. Hence, our observations indicate that the compositional distribution of the core-shell heterostructures is dominated by the thermodynamic preferences of their constituents.

**Figure 6:**
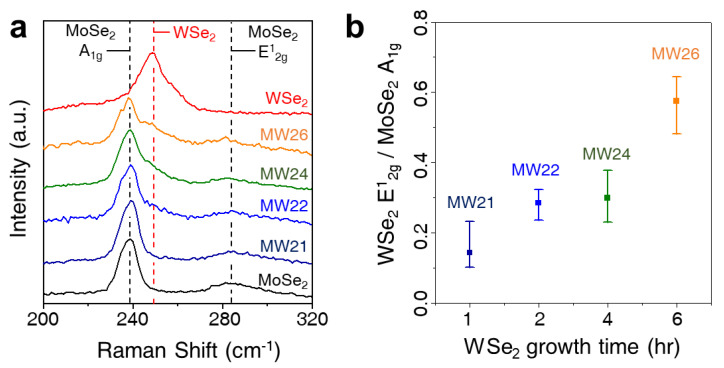
(**a**) Raman spectra of MoSe_2_/WSe_2_ heterostructures (MW21, MW22, MW24, and MW26), shown together with the data of pure MoSe_2_ and WSe_2_. The vertical dashed black lines correspond to the MoSe_2_ A_1g_ (239 cm^−1^) and E^1^_2g_ (283 cm^−1^) peaks. The vertical dashed red line denotes the WSe_2_ E^1^_2g_ (249 cm^−1^) peak. (**b**) Raman intensity ratio of WSe_2_ E^1^_2g_ to MoSe_2_ A_1g_ as a function of the secondary WSe_2_ growth time.

Figure 6a shows the Raman spectra of the core-shell heterostructures prepared with various MoSe_2_ secondary growth times. The Raman peaks of the pure MoSe_2_ nanosheet (black) are located at 240 and 283 cm^−1^, corresponding to the out-of-plane A_1g_ and in-plane E^1^_2g_ modes, respectively, whereas the WSe_2_ nanosheet (red) exhibits a degenerated E^1^_2g_-like peak centered at 249 cm^−1^ [26,27]. While the hetero-nanosheets (MW21, MW22, MW24, and MW26) displayed Raman spectra largely similar to the core-only MoSe_2_, the emergence of the shoulder peaks at 249 cm^−1^ and their increase in intensity were evident as the secondary growth time increased from 1 h to 6 h. Notably, the MoSe_2_ peaks of the heterostructures did not exhibit a shift in wavenumbers, suggesting that the WSe_2_ shell growth did not affect the lattice strain of the MoSe_2_ core. Furthermore, the typical features of the A_1g_ blue-shift and E^1^_2g_ red-shift corresponding to the formation of Mo_x_W_1−x_Se_2_ alloy were absent [28], supporting the core-shell-like structure for all the samples. To confirm the compositional dependence of the heterostructures on the secondary growth time, the Raman intensity ratio of the characteristic E^1^_2g_ peak of WSe_2_ (249 cm^−1^) to MoSe_2_ (240 cm^−1^) was plotted as a function of WSe_2_ growth time, as illustrated in Figure 6b. The tendency of the asymptotic increase in the intensity ratio with the secondary growth time confirmed the tunable overgrowth of the WSe_2_ shell, which is immiscible with the MoSe_2_ core.

**Figure 7:**
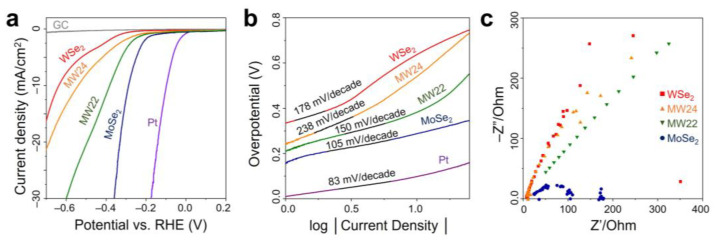
(**a**) Linear sweep voltammetry (LSV) analysis conducted on Pt, MoSe_2_, MW22, MW24, WSe_2_, and GC (glassy carbon) electrodes. (**b**) Tafel slopes corresponding to the LSV analysis shown in (**a**). (**c**) Nyquist plots obtained from the electrochemical impedance spectroscopy (EIS) measurements.

The obtained MoSe_2_/WSe_2_ core-shell heterostructures with variable shell thicknesses were further applied to the HER electrochemical reaction. We compared the HER performance of the as-grown heterostructures using a three-electrode configuration, as shown in Figure 7. Figure 7a shows the linear sweep voltammetry (LSV) curves of MW22 and MW24 transferred onto glassy carbon (GC) electrodes. The data from pure MoSe_2_ and WSe_2_ are shown together with the Pt electrode, as a reference. The overpotential values at a current density of 10 mA/cm^2^ were 80, 340, 410, 580, and 650 mV for Pt, MoSe_2_, MW22, MW24, and WSe_2_, respectively.

While MoSe_2_ required a lower overpotential than WSe_2_, the heterostructures exhibited intermediate performance according to the WSe_2_ shell growth time. The corresponding Tafel slopes (Figure 7b) of 83 (Pt), 105 (MoSe_2_), 150 (MW22), 238 (MW24), and 178 (WSe_2_) mV/decade additionally reflected the relative HER performance. Finally, the Nyquist plot obtained from the electrochemical impedance spectroscopy (EIS) shown in Figure 7c further confirmed that the resistance of MW22 (580 Ω) and MW24 (713 Ω) is between that of MoSe_2_ (86 Ω) and WSe_2_ (1652 Ω). Table 1 summarizes the electrochemical performances of the colloidal TMDs prepared in this study compared with previously reported data. While the absolute performance values are inferior to the published values, presumably owing to the presence of residual precursors, our results provide important implications regarding the dependence of electrochemical activity on structural characteristics of MoSe_2_/WSe_2_ core-shell heterostructures. Given that the edges of MoSe_2_ exhibit lower hydrogen adsorption energy relative to WSe_2_, the increase in the shell growth time would result in the reduction of MoSe_2_ edges and a subsequent decrease in the electrochemical activity. Thus, the intermediate HER performances of heterostructures, dependent on the secondary growth time, suggest that the core-shell growth protocol proposed in this study can be applied to tune the electrochemical activities of colloidal TMD materials.

## 4. Conclusions

We presented core-shell heterostructures of MoSe_2_/WSe_2_ TMDs obtained via a two-step colloidal synthesis. Comprehensive structural and chemical analyses confirmed that the as-grown heterostructures were composed of a spatially distinct 2H-MoSe_2_ core and WSe_2_ shell layers. Due to the lower regime of synthetic temperatures relative to the vapor-phase-based approach, the colloidal growth of TMDs in solution enables the formation of phase-segregated, core-shell-like structures rather than uniformly distributed alloys. The composition of the shell layer was tunable by changing the secondary growth time, and the spatial distribution of constituents in the heterostructure was thermodynamically determined as such MoSe_2_ resides in the core region. Notably, the electrochemical properties measured from the as-grown samples correlated to the variety of core-shell compositions. Our results offer an opportunity to control the properties of colloidal TMDs, depending on the edge structure. The ability to modulate electrochemistry by engineering active edge sites is critical for developing highly efficient HER electrocatalysts.

## Figures and Tables

**Table 1:** HER performance comparison of MoSe_2_-based nanostructure catalysts showing the overpotentials and Tafel slopes.

Materials	Loading	η at j = 10 mA/cm^2^ (mV)	Tafel Slope (mV/decade)	Ref.
MoSe_2_ nanoflowers	0.28 mg cm^−2^ on GC	340	105	This work
MW22 nanoflowers		410	150	
MW24 nanoflowers		580	238	
WSe_2_ nanoflowers		650	178	
Bulk MoSe_2_	0.28 mg cm^−2^ on GC	>600	346	[14]
Porous MoSe_2_ nanosheets	0.47 mg cm^−2^ on GC	150	80	[29]
Vertically aligned MoSe_2_ layers	0.0135 mg cm^−2^ on GC	−450	105–120	[30]
Hierarchical MoSe_2−x_ nanosheets	0.28 mg cm^−2^ on GC	288	98	[14]
MoSe_2_ nanoflakes (2:1 ratio to Ketjen black)	0.28 mg cm^−2^ on GC	181 ± 1	45 ± 1	[31]
MoSe_2_ nanonetworks (8:1 ratio to Ketjen black)	0.28 mg cm^−2^ on GC	302	109	[19]
MoSe_2_ nanoflowers (8:1 ratio to Ketjen black)	0.28 mg cm^−2^ on GC	233	89	[19]

## Data Availability

The data presented in this study are available in this article.
